# Supplementary material for: Bangla MedER: Multi-BERT ensemble approach for the recognition of Bangla medical entity
Source: PLoS One. 2026 Feb 26;21(2):e0342558. doi: 10.1371/journal.pone.0342558 (PMC12944717; doi:10.1371/journal.pone.0342558)
Supplement: Attachment — Submitted filename: Response to the Reviewer MedER.docx [file pone.0342558.s001.docx]

**Response to the Reviewer:**

**Editor**

1. Include quantitative impact in both the abstract and conclusion, and add a subsection addressing limitations, challenges, and contributions.
2. **Response:** We have addressed this comment as follows:

- The **quantitative impact** has been highlighted in both the abstract and conclusion. For example, we explicitly state: “The proposed model outperforms existing transformer-based models, achieving an 11.80% accuracy improvement over a single-layer BERT model.”
- A new subsection titled “**5.1 Limitations, Challenges, and Contributions**” has been added to clearly discuss the limitations of our approach, the challenges faced during development, and the key contributions of our work.

3. Summarize the literature review in a comparative table, provide more details on datasets and experimental setup, and increase the number of references to strengthen the scholarly foundation.

**Response:** The Related Work section has been updated accordingly. Details about datasets and experimental setup have been updated.

4. Clearly indicate how data were extracted, ensure compliance with PLOS ONE’s code and dataset availability requirements.
5. Despite the inclusion of Section 7 on ethical statements, further enhancements are required for PLOS ONE compliance include clear statement on ethical considerations related to data collection and usage.

**Reviewer 2**

1. The introduction is very simple and needs further expansion to clarify the research gap and what researchers in this field are seeking.

**Response:** The Introduction section has been updated accordingly.

2. Sort research papers in Related Work by year of publication (from oldest to newest) and add research papers published in 2025. It is also preferable to add a table summarizing the pros and cons of each included research paper.

**Response:** The Related Work section has been updated accordingly. Papers are now sorted chronologically from old to newest, and a table summarizing the pros and cons of each included research paper has been added.

3. Comparison of the submitted work with other research papers published in the years 2023-2025

**Response:** The manuscript has been revised to include a comparison of the submitted work with relevant research published between 2023 and 2025.

4. Adding recent references published in 2025, with no less than three references

**Response:** Four references from 2025 have been incorporated into the manuscript.

| **Author** | **Year** | **Domain/Focus** | **Pros** | **Cons** |
| --- | --- | --- | --- | --- |
| Ashrafi et al. | 2020 | General Bangla NER | Pioneering cost-sensitive BiLSTM-CRF for Bangla Handles severe class imbalance Open-source code | Pre-transformer architecture Outperformed by later models Not domain-specific |
| Ge et al. | 2020 | Medical NER + Federated Learning | First federated learning approach for medical NER Preserves patient privacy | Lower performance than centralized models High communication cost Early 2020 design |
| Gligic et al. | 2020 | EHR NER (English) | Strong transfer-learning + bootstrapped nets on i2b2 datasets | English only Older architecture (2020) |
| Perera et al. | 2020 | Biomedical NER + Relation Extraction | Useful joint NER+RE model + overview | Partly survey-like No longer SOTA |
| Catelli et al. | 2020 | Clinical de-identification (Italian COVID) | Effective cross-lingual transfer for low-resource Italian clinical text | Italian only Limited to early COVID-19 data |
| Liu et al. | 2021 | Medical NER (mainly Chinese) | First successful domain-specific pretraining on large medical corpora Big gains over general BERT | Primarily Chinese EHRs Requires further adaptation for other languages (incl. Bangla) |
| Haque et al. | 2023 | General Bangla NER dataset | Largest Bangla NER dataset (~300k tokens, 19 entity types) Diverse sources Public + baselines | General domain Some annotation inconsistencies noted later Baselines now outdated |
| Muntakim et al. | 2023 | Bangla Medical NER corpus | First gold-standard Bangla medical NER corpus Expert-annotated Critical for Bangla healthcare NLP | Very small (~6k sentences) Only 5 entity types Baselines relatively weak |
| Lima et al. | 2023 | General Bengali NER | Data-centric + model-centric hybrid Reached ~94% F1 at the time Synthetic data + ensemble | Complex and hard to reproduce Not specialized Since surpassed by newer models |
| Shahgir et al. | 2023 | Bangla Complex/Nested NER | First model targeting complex & nested entities in Bangla Strong on nested structures | Still arXiv preprint Higher complexity Weaker on flat entities |
| Aurpa et al. | 2024 | Bangla Mathematical Entity dataset | First (and only) public Bangla mathematical entity dataset Carefully annotated math expressions | Niche domain Relatively small (~20k sentences) |
| Aurpa & Ahmed | 2024 | Bangla Mathematical Entity Recognition | First dedicated Bangla MER system Novel ensemble (BanglaBERT + mBERT + XLM-R + CNN-BiLSTM) ~94–96% F1 | Math-only domain Ensemble increases latency & complexity Depends on their own dataset |
| Farhan et al. | 2024 | General Bangla NER | Creative gazetteer + semantic clustering in CRF Significant gains over plain BanglaBERT | Heavy gazetteer dependence (may hurt generalization) Still arXiv Complex pipeline |
| García-Barragán et al. | 2025 | Medical NER (Spanish) | Leverages GPT-4 / LLMs for very high performance on Spanish clinical texts | High cost & latency Risk of hallucinations Not open-weight |
| Yan et al. | 2025 | Chinese Medical NER | Entity-association + gate context awareness New SOTA on Chinese clinical datasets | Chinese only Complex architecture |
| Wei et al. | 2025 | General Medical NER | Focuses on efficiency in the “Silicon Revolution” era Claims strong results | Very recent → limited independent validation Likely large-scale model |
| Sun et al. | 2025 | Medical NER (domain knowledge + position) | Integrates external medical knowledge + improved position encoding Strong gains on multiple datasets | Increased complexity Requires external knowledge base |
