# Supplementary material for: Bangla MedER: Multi-BERT ensemble approach for the recognition of Bangla medical entity
Source: PLoS One. 2026 Feb 26;21(2):e0342558. doi: 10.1371/journal.pone.0342558 (PMC12944717; doi:10.1371/journal.pone.0342558)
Supplement: Attachment — Submitted filename: Response Letter.docx [file pone.0342558.s002.docx]

**ID:** PONE-D-25-12318R1

**Title:** Bangla MedER: Multi-BERT Ensemble Approach for the Recognition of Bangla Medical Entity

**Code Sharing & Reproducibility:** In accordance with our reproducibility guidelines, please provide the specific source code or scripts used to generate the results presented in Table 5 and Figures 6 and 7. We recommend depositing this code in a public repository (e.g., GitHub, Zenodo, or Figshare) and providing the DOI or link within your Data Availability Statement.

**Response:** The complete implementation can be accessed through the **Data and Code Availability** section of the manuscript, which provides the corresponding repository link.

**Data Alignment:** There appear to be discrepancies between the figures and their corresponding tables. Please ensure that all data points are fully synchronized across Table 5, Figure 6, and Figure 7.

**Response:** There is no Table 5 in the manuscript; the comment likely refers to Table 3. Upon careful review, we identified a minor typographical error which has now been corrected, and the data across Table 3, Figure 6, and Figure 7 are fully synchronized.

**Comprehensive Metrics:** The current manuscript does not provide a complete set of class label metrics for the top-performing model. Please update the results section to include a granular breakdown (e.g., precision, recall, and F1-score for each class) to allow for a thorough validation of the model's performance.

**Response:** The class-wise performance metrics for the top-performing model are reported in Table 3. Figure 7 presents an overall performance comparison of the models, offering a complementary summary of the results.
